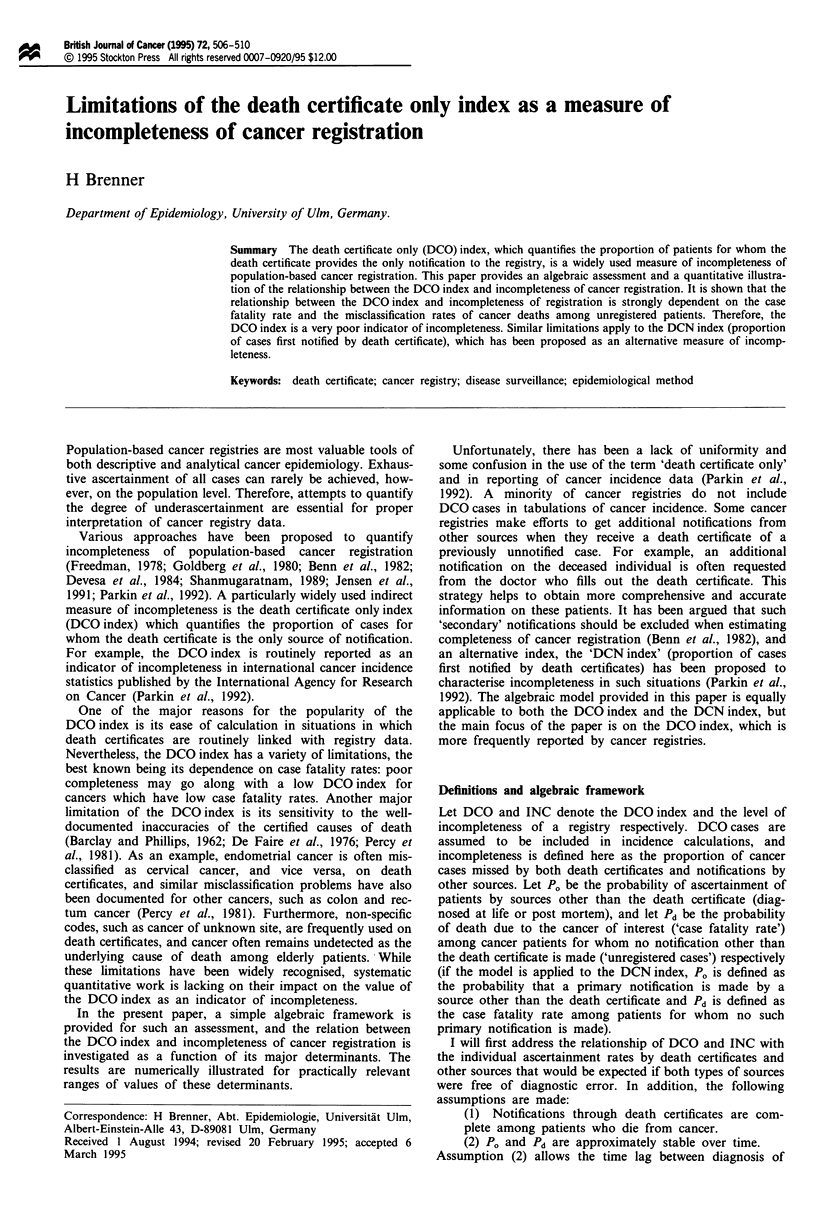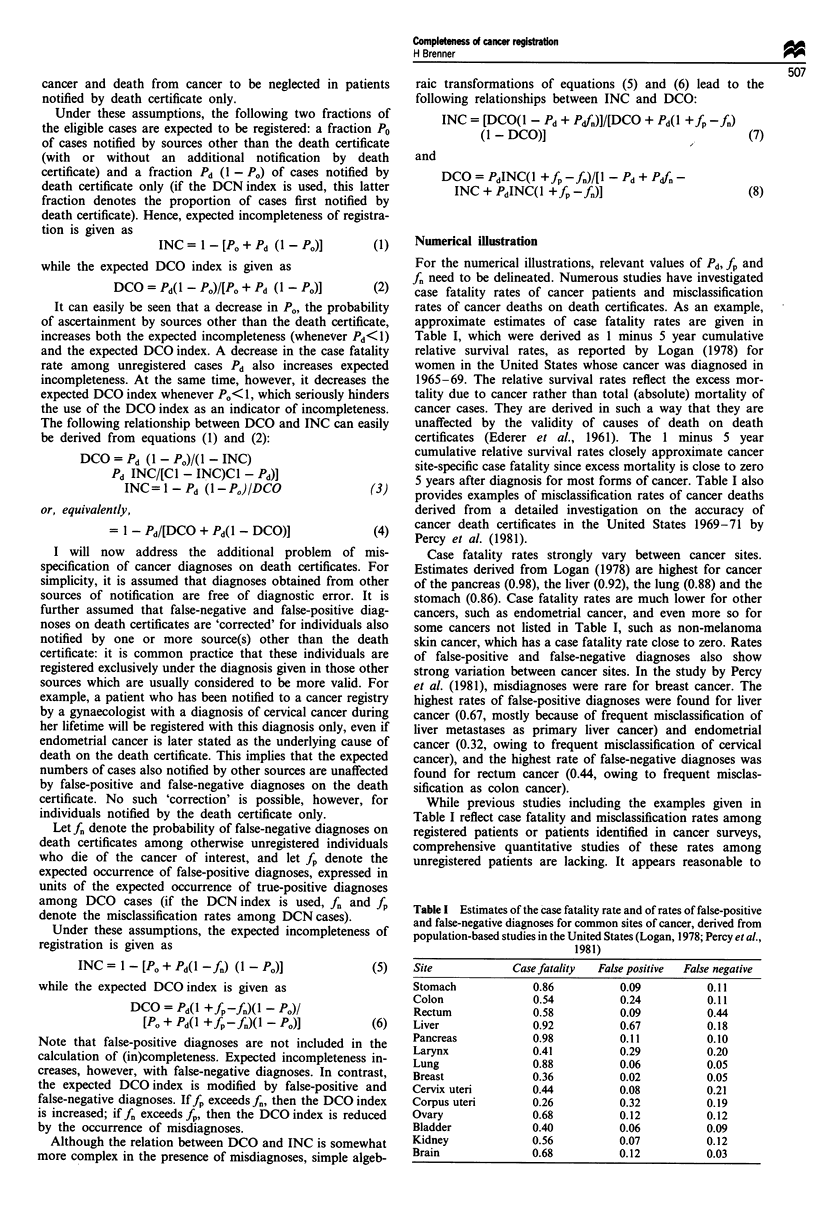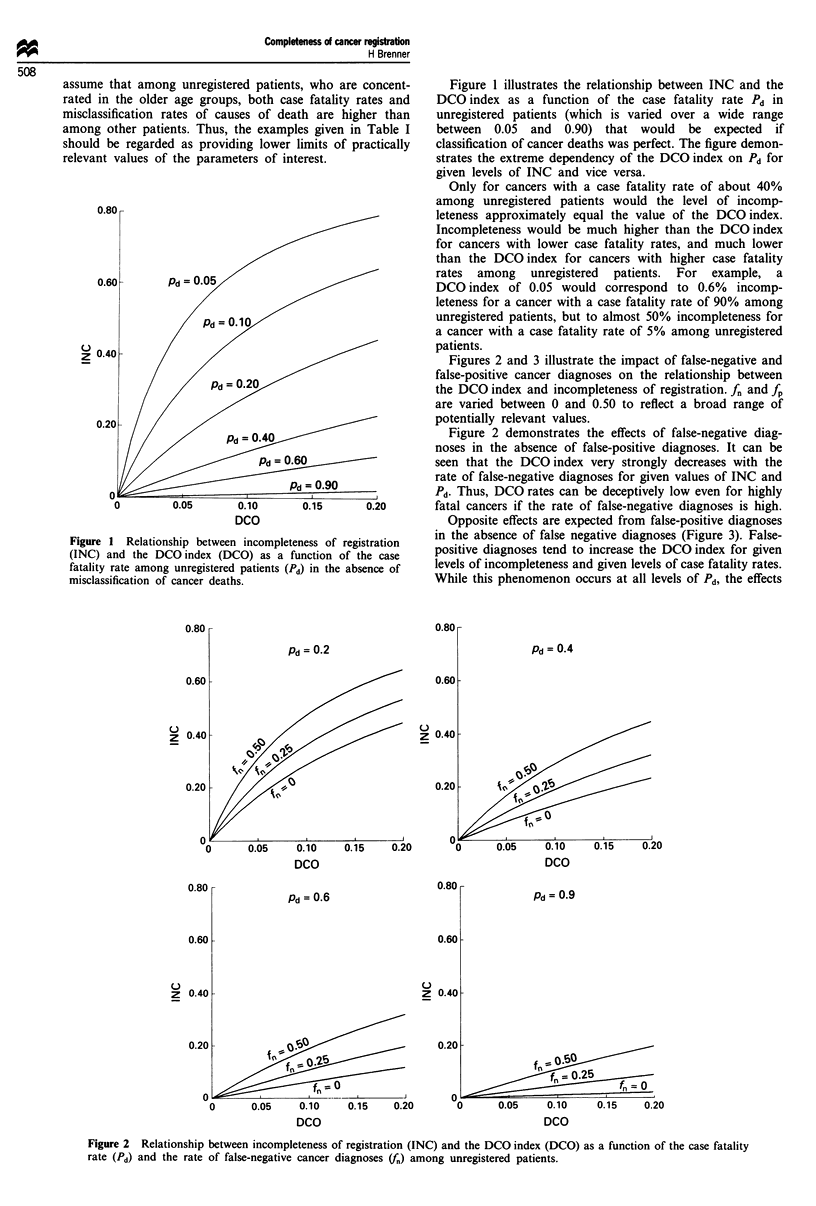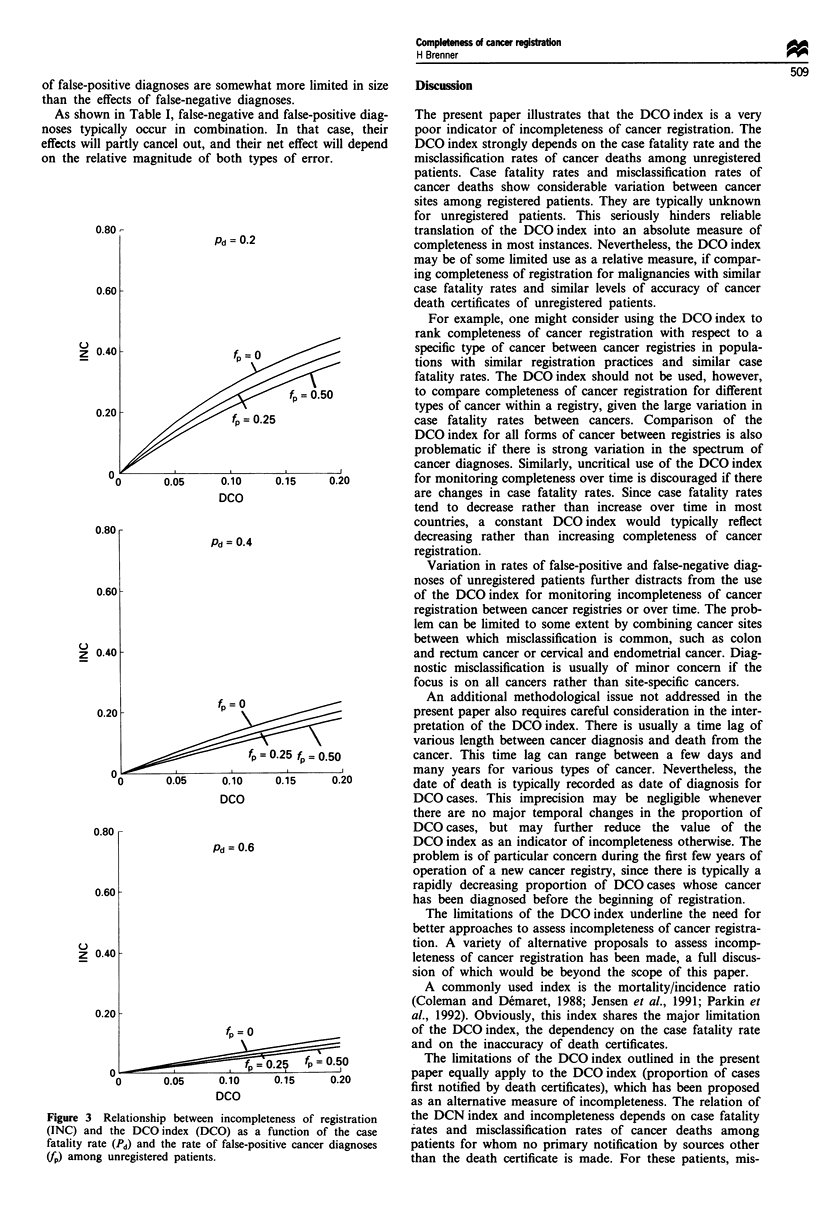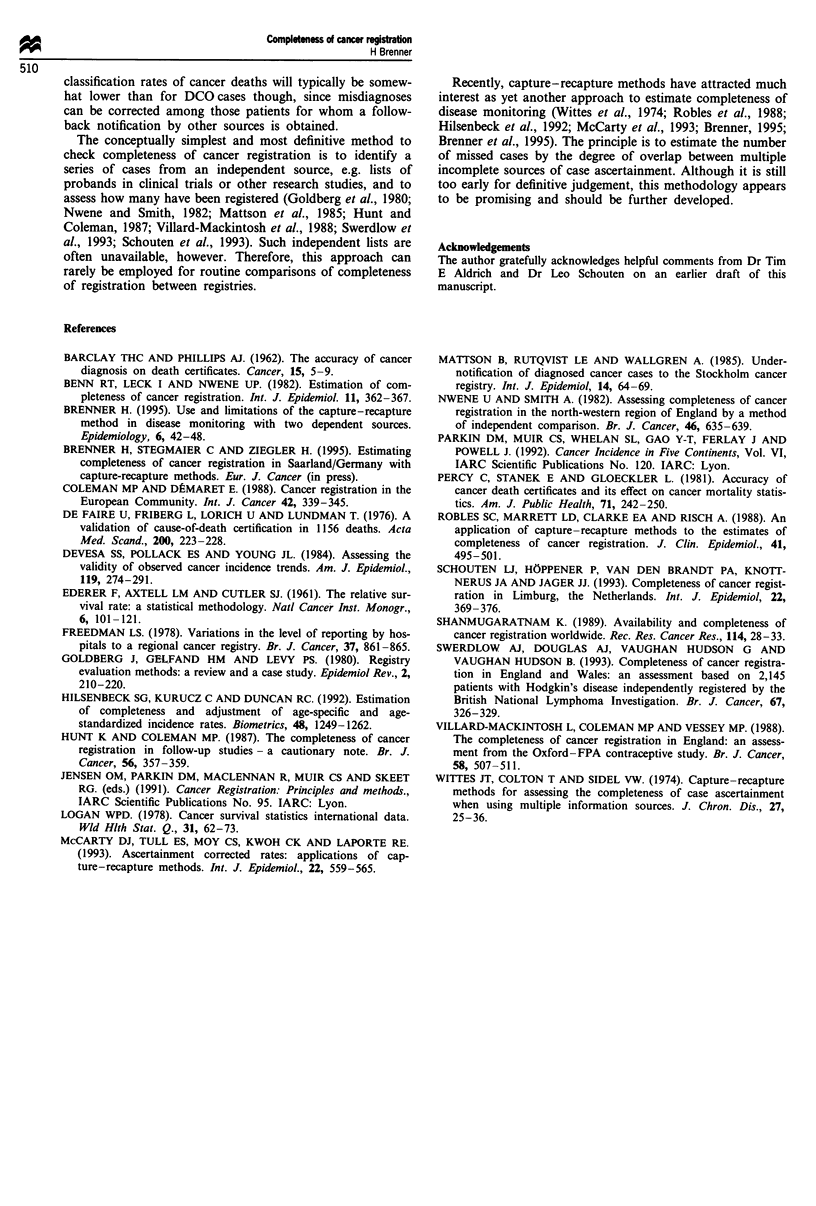# Limitations of the death certificate only index as a measure of incompleteness of cancer registration.

**DOI:** 10.1038/bjc.1995.363

**Published:** 1995-08

**Authors:** H. Brenner

**Affiliations:** Department of Epidemiology, University of Ulm, Germany.

## Abstract

The death certificate only (DCO) index, which quantifies the proportion of patients for whom the death certificate provides the only notification to the registry, is a widely used measure of incompleteness of population-based cancer registration. This paper provides an algebraic assessment and a quantitative illustration of the relationship between the DCO index and incompleteness of cancer registration. It is shown that the relationship between the DCO index and incompleteness of registration is strongly dependent on the case fatality rate and the misclassification rates of cancer deaths among unregistered patients. Therefore, the DCO index is a very poor indicator of incompleteness. Similar limitations apply to the DCN index (proportion of cases first notified by death certificate), which has been proposed as an alternative measure of incompleteness.


British Journal of Cancer (1995) 72, 506–510
© 1995 Stockton Press All rights reserved 0007–0920/95 $12.00

### Limitations of the death certificate only index as a measure of incompleteness of cancer registration

H Brenner

*Department of Epidemiology, University of Ulm, Germany.*

**Summary** The death certificate only (DCO) index, which quantifies the proportion of patients for whom the death certificate provides the only notification to the registry, is a widely used measure of incompleteness of population-based cancer registration. This paper provides an algebraic assessment and a quantitative illustration of the relationship between the DCO index and incompleteness of cancer registration. It is shown that the relationship between the DCO index and incompleteness of registration is strongly dependent on the case fatality rate and the misclassification rates of cancer deaths among unregistered patients. Therefore, the DCO index is a very poor indicator of incompleteness. Similar limitations apply to the DCN index (proportion of cases first notified by death certificate), which has been proposed as an alternative measure of incompleteness.

**Keywords:** death certificate; cancer registry; disease surveillance; epidemiological method

Population-based cancer registries are most valuable tools of both descriptive and analytical cancer epidemiology. Exhaustive ascertainment of all cases can rarely be achieved, however, on the population level. Therefore, attempts to quantify the degree of underascertainment are essential for proper interpretation of cancer registry data.

Various approaches have been proposed to quantify incompleteness of population-based cancer registration (Freedman, 1978; Goldberg *et al.*, 1980; Benn *et al.*, 1982; Devesa *et al.*, 1984; Shanmugaratnam, 1989; Jensen *et al.*, 1991; Parkin *et al.*, 1992). A particularly widely used indirect measure of incompleteness is the death certificate only index (DCO index) which quantifies the proportion of cases for whom the death certificate is the only source of notification. For example, the DCO index is routinely reported as an indicator of incompleteness in international cancer incidence statistics published by the International Agency for Research on Cancer (Parkin *et al.*, 1992).

One of the major reasons for the popularity of the DCO index is its ease of calculation in situations in which death certificates are routinely linked with registry data. Nevertheless, the DCO index has a variety of limitations, the best known being its dependence on case fatality rates: poor completeness may go along with a low DCO index for cancers which have low case fatality rates. Another major limitation of the DCO index is its sensitivity to the well-documented inaccuracies of the certified causes of death (Barclay and Phillips, 1962; De Faire *et al.*, 1976; Percy *et al.*, 1981). As an example, endometrial cancer is often misclassified as cervical cancer, and vice versa, on death certificates, and similar misclassification problems have also been documented for other cancers, such as colon and rectum cancer (Percy *et al.*, 1981). Furthermore, non-specific codes, such as cancer of unknown site, are frequently used on death certificates, and cancer often remains undetected as the underlying cause of death among elderly patients. While these limitations have been widely recognised, systematic quantitative work is lacking on their impact on the value of the DCO index as an indicator of incompleteness.

In the present paper, a simple algebraic framework is provided for such an assessment, and the relation between the DCO index and incompleteness of cancer registration is investigated as a function of its major determinants. The results are numerically illustrated for practically relevant ranges of values of these determinants.

Correspondence: H Brenner, Abt. Epidemiologie, Universität Ulm, Albert-Einstein-Alle 43, D-89081 Ulm, Germany
Received 1 August 1994; revised 20 February 1995; accepted 6 March 1995

Unfortunately, there has been a lack of uniformity and some confusion in the use of the term 'death certificate only' and in reporting of cancer incidence data (Parkin *et al.*, 1992). A minority of cancer registries do not include DCO cases in tabulations of cancer incidence. Some cancer registries make efforts to get additional notifications from other sources when they receive a death certificate of a previously unnotified case. For example, an additional notification on the deceased individual is often requested from the doctor who fills out the death certificate. This strategy helps to obtain more comprehensive and accurate information on these patients. It has been argued that such 'secondary' notifications should be excluded when estimating completeness of cancer registration (Benn *et al.*, 1982), and an alternative index, the 'DCN index' (proportion of cases first notified by death certificates) has been proposed to characterise incompleteness in such situations (Parkin *et al.*, 1992). The algebraic model provided in this paper is equally applicable to both the DCO index and the DCN index, but the main focus of the paper is on the DCO index, which is more frequently reported by cancer registries.

### Definitions and algebraic framework

Let DCO and INC denote the DCO index and the level of incompleteness of a registry respectively. DCO cases are assumed to be included in incidence calculations, and incompleteness is defined here as the proportion of cancer cases missed by both death certificates and notifications by other sources. Let $P_o$ be the probability of ascertainment of patients by sources other than the death certificate (diagnosed at life or post mortem), and let $P_d$ be the probability of death due to the cancer of interest ('case fatality rate') among cancer patients for whom no notification other than the death certificate is made ('unregistered cases') respectively (if the model is applied to the DCN index, $P_o$ is defined as the probability that a primary notification is made by a source other than the death certificate and $P_d$ is defined as the case fatality rate among patients for whom no such primary notification is made).

I will first address the relationship of DCO and INC with the individual ascertainment rates by death certificates and other sources that would be expected if both types of sources were free of diagnostic error. In addition, the following assumptions are made:

(1) Notifications through death certificates are complete among patients who die from cancer.

(2) $P_o$ and $P_d$ are approximately stable over time.

Assumption (2) allows the time lag between diagnosis of cancer and death from cancer to be neglected in patients notified by death certificate only.

Under these assumptions, the following two fractions of the eligible cases are expected to be registered: a fraction $P_0$ of cases notified by sources other than the death certificate (with or without an additional notification by death certificate) and a fraction $P_d$ $(1 - P_o)$ of cases notified by death certificate only (if the DCN index is used, this latter fraction denotes the proportion of cases first notified by death certificate). Hence, expected incompleteness of registration is given as

$$\text{INC} = 1 - [P_o + P_d\ (1 - P_o)] \qquad (1)$$

while the expected DCO index is given as

$$\text{DCO} = P_d(1 - P_o)/[P_o + P_d\ (1 - P_o)] \qquad (2)$$

It can easily be seen that a decrease in $P_o$, the probability of ascertainment by sources other than the death certificate, increases both the expected incompleteness (whenever $P_d<1$) and the expected DCO index. A decrease in the case fatality rate among unregistered cases $P_d$ also increases expected incompleteness. At the same time, however, it decreases the expected DCO index whenever $P_o<1$, which seriously hinders the use of the DCO index as an indicator of incompleteness. The following relationship between DCO and INC can easily be derived from equations (1) and (2):

$$\text{DCO} = P_d\ (1 - P_o)/(1 - \text{INC})$$

$$P_d\ \text{INC}/[\text{C}1 - \text{INC})\text{C}1 - P_d)]$$

$$\text{INC} = 1 - P_d\ (1 - P_o)/DCO \qquad (3)$$

*or, equivalently,*

$$= 1 - P_d/[\text{DCO} + P_d(1 - \text{DCO})] \qquad (4)$$

I will now address the additional problem of misspecification of cancer diagnoses on death certificates. For simplicity, it is assumed that diagnoses obtained from other sources of notification are free of diagnostic error. It is further assumed that false-negative and false-positive diagnoses on death certificates are 'corrected' for individuals also notified by one or more source(s) other than the death certificate: it is common practice that these individuals are registered exclusively under the diagnosis given in those other sources which are usually considered to be more valid. For example, a patient who has been notified to a cancer registry by a gynaecologist with a diagnosis of cervical cancer during her lifetime will be registered with this diagnosis only, even if endometrial cancer is later stated as the underlying cause of death on the death certificate. This implies that the expected numbers of cases also notified by other sources are unaffected by false-positive and false-negative diagnoses on the death certificate. No such 'correction' is possible, however, for individuals notified by the death certificate only.

Let $f_n$ denote the probability of false-negative diagnoses on death certificates among otherwise unregistered individuals who die of the cancer of interest, and let $f_p$ denote the expected occurrence of false-positive diagnoses, expressed in units of the expected occurrence of true-positive diagnoses among DCO cases (if the DCN index is used, $f_n$ and $f_p$ denote the misclassification rates among DCN cases).

Under these assumptions, the expected incompleteness of registration is given as

$$\text{INC} = 1 - [P_o + P_d(1 - f_n)\ (1 - P_o)] \qquad (5)$$

while the expected DCO index is given as

$$\text{DCO} = P_d(1 + f_p - f_n)(1 - P_o)/$$
$$[P_o + P_d(1 + f_p - f_n)(1 - P_o)] \qquad (6)$$

Note that false-positive diagnoses are not included in the calculation of (in)completeness. Expected incompleteness increases, however, with false-negative diagnoses. In contrast, the expected DCO index is modified by false-positive and false-negative diagnoses. If $f_p$ exceeds $f_n$, then the DCO index is increased; if $f_n$ exceeds $f_p$, then the DCO index is reduced by the occurrence of misdiagnoses.

Although the relation between DCO and INC is somewhat more complex in the presence of misdiagnoses, simple algebraic transformations of equations (5) and (6) lead to the following relationships between INC and DCO:

$$\text{INC} = [\text{DCO}(1 - P_d + P_d f_n)]/[\text{DCO} + P_d(1 + f_p - f_n)$$
$$(1 - \text{DCO})] \qquad (7)$$

and

$$\text{DCO} = P_d\text{INC}(1 + f_p - f_n)/[1 - P_d + P_d f_n -$$

$$\text{INC} + P_d\text{INC}(1 + f_p - f_n)] \qquad (8)$$

### Numerical illustration

For the numerical illustrations, relevant values of $P_d$, $f_p$ and $f_n$ need to be delineated. Numerous studies have investigated case fatality rates of cancer patients and misclassification rates of cancer deaths on death certificates. As an example, approximate estimates of case fatality rates are given in Table I, which were derived as 1 minus 5 year cumulative relative survival rates, as reported by Logan (1978) for women in the United States whose cancer was diagnosed in 1965–69. The relative survival rates reflect the excess mortality due to cancer rather than total (absolute) mortality of cancer cases. They are derived in such a way that they are unaffected by the validity of causes of death on death certificates (Ederer *et al.*, 1961). The 1 minus 5 year cumulative relative survival rates closely approximate cancer site-specific case fatality since excess mortality is close to zero 5 years after diagnosis for most forms of cancer. Table I also provides examples of misclassification rates of cancer deaths derived from a detailed investigation on the accuracy of cancer death certificates in the United States 1969–71 by Percy *et al.* (1981).

Case fatality rates strongly vary between cancer sites. Estimates derived from Logan (1978) are highest for cancer of the pancreas (0.98), the liver (0.92), the lung (0.88) and the stomach (0.86). Case fatality rates are much lower for other cancers, such as endometrial cancer, and even more so for some cancers not listed in Table I, such as non-melanoma skin cancer, which has a case fatality rate close to zero. Rates of false-positive and false-negative diagnoses also show strong variation between cancer sites. In the study by Percy *et al.* (1981), misdiagnoses were rare for breast cancer. The highest rates of false-positive diagnoses were found for liver cancer (0.67, mostly because of frequent misclassification of liver metastases as primary liver cancer) and endometrial cancer (0.32, owing to frequent misclassification of cervical cancer), and the highest rate of false-negative diagnoses was found for rectum cancer (0.44, owing to frequent misclassification as colon cancer).

While previous studies including the examples given in Table I reflect case fatality and misclassification rates among registered patients or patients identified in cancer surveys, comprehensive quantitative studies of these rates among unregistered patients are lacking. It appears reasonable to

**Table I** Estimates of the case fatality rate and of rates of false-positive and false-negative diagnoses for common sites of cancer, derived from population-based studies in the United States (Logan, 1978; Percy *et al.*, 1981)

| *Site* | *Case fatality* | *False positive* | *False negative* |
|---|---|---|---|
| Stomach | 0.86 | 0.09 | 0.11 |
| Colon | 0.54 | 0.24 | 0.11 |
| Rectum | 0.58 | 0.09 | 0.44 |
| Liver | 0.92 | 0.67 | 0.18 |
| Pancreas | 0.98 | 0.11 | 0.10 |
| Larynx | 0.41 | 0.29 | 0.20 |
| Lung | 0.88 | 0.06 | 0.05 |
| Breast | 0.36 | 0.02 | 0.05 |
| Cervix uteri | 0.44 | 0.08 | 0.21 |
| Corpus uteri | 0.26 | 0.32 | 0.19 |
| Ovary | 0.68 | 0.12 | 0.12 |
| Bladder | 0.40 | 0.06 | 0.09 |
| Kidney | 0.56 | 0.07 | 0.12 |
| Brain | 0.68 | 0.12 | 0.03 |

assume that among unregistered patients, who are concentrated in the older age groups, both case fatality rates and misclassification rates of causes of death are higher than among other patients. Thus, the examples given in Table I should be regarded as providing lower limits of practically relevant values of the parameters of interest.

**Figure 1** Relationship between incompleteness of registration (INC) and the DCO index (DCO) as a function of the case fatality rate among unregistered patients ($P_d$) in the absence of misclassification of cancer deaths.

Figure 1 illustrates the relationship between INC and the DCO index as a function of the case fatality rate $P_d$ in unregistered patients (which is varied over a wide range between 0.05 and 0.90) that would be expected if classification of cancer deaths was perfect. The figure demonstrates the extreme dependency of the DCO index on $P_d$ for given levels of INC and vice versa.

Only for cancers with a case fatality rate of about 40% among unregistered patients would the level of incompleteness approximately equal the value of the DCO index. Incompleteness would be much higher than the DCO index for cancers with lower case fatality rates, and much lower than the DCO index for cancers with higher case fatality rates among unregistered patients. For example, a DCO index of 0.05 would correspond to 0.6% incompleteness for a cancer with a case fatality rate of 90% among unregistered patients, but to almost 50% incompleteness for a cancer with a case fatality rate of 5% among unregistered patients.

Figures 2 and 3 illustrate the impact of false-negative and false-positive cancer diagnoses on the relationship between the DCO index and incompleteness of registration. $f_n$ and $f_p$ are varied between 0 and 0.50 to reflect a broad range of potentially relevant values.

Figure 2 demonstrates the effects of false-negative diagnoses in the absence of false-positive diagnoses. It can be seen that the DCO index very strongly decreases with the rate of false-negative diagnoses for given values of INC and $P_d$. Thus, DCO rates can be deceptively low even for highly fatal cancers if the rate of false-negative diagnoses is high.

Opposite effects are expected from false-positive diagnoses in the absence of false negative diagnoses (Figure 3). False-positive diagnoses tend to increase the DCO index for given levels of incompleteness and given levels of case fatality rates. While this phenomenon occurs at all levels of $P_d$, the effects

**Figure 2** Relationship between incompleteness of registration (INC) and the DCO index (DCO) as a function of the case fatality rate ($P_d$) and the rate of false-negative cancer diagnoses ($f_n$) among unregistered patients.

of false-positive diagnoses are somewhat more limited in size than the effects of false-negative diagnoses.

As shown in Table I, false-negative and false-positive diagnoses typically occur in combination. In that case, their effects will partly cancel out, and their net effect will depend on the relative magnitude of both types of error.

**Figure 3** Relationship between incompleteness of registration (INC) and the DCO index (DCO) as a function of the case fatality rate ($P_d$) and the rate of false-positive cancer diagnoses ($f_p$) among unregistered patients.

### Discussion

The present paper illustrates that the DCO index is a very poor indicator of incompleteness of cancer registration. The DCO index strongly depends on the case fatality rate and the misclassification rates of cancer deaths among unregistered patients. Case fatality rates and misclassification rates of cancer deaths show considerable variation between cancer sites among registered patients. They are typically unknown for unregistered patients. This seriously hinders reliable translation of the DCO index into an absolute measure of completeness in most instances. Nevertheless, the DCO index may be of some limited use as a relative measure, if comparing completeness of registration for malignancies with similar case fatality rates and similar levels of accuracy of cancer death certificates of unregistered patients.

For example, one might consider using the DCO index to rank completeness of cancer registration with respect to a specific type of cancer between cancer registries in populations with similar registration practices and similar case fatality rates. The DCO index should not be used, however, to compare completeness of cancer registration for different types of cancer within a registry, given the large variation in case fatality rates between cancers. Comparison of the DCO index for all forms of cancer between registries is also problematic if there is strong variation in the spectrum of cancer diagnoses. Similarly, uncritical use of the DCO index for monitoring completeness over time is discouraged if there are changes in case fatality rates. Since case fatality rates tend to decrease rather than increase over time in most countries, a constant DCO index would typically reflect decreasing rather than increasing completeness of cancer registration.

Variation in rates of false-positive and false-negative diagnoses of unregistered patients further distracts from the use of the DCO index for monitoring incompleteness of cancer registration between cancer registries or over time. The problem can be limited to some extent by combining cancer sites between which misclassification is common, such as colon and rectum cancer or cervical and endometrial cancer. Diagnostic misclassification is usually of minor concern if the focus is on all cancers rather than site-specific cancers.

An additional methodological issue not addressed in the present paper also requires careful consideration in the interpretation of the DCO index. There is usually a time lag of various length between cancer diagnosis and death from the cancer. This time lag can range between a few days and many years for various types of cancer. Nevertheless, the date of death is typically recorded as date of diagnosis for DCO cases. This imprecision may be negligible whenever there are no major temporal changes in the proportion of DCO cases, but may further reduce the value of the DCO index as an indicator of incompleteness otherwise. The problem is of particular concern during the first few years of operation of a new cancer registry, since there is typically a rapidly decreasing proportion of DCO cases whose cancer has been diagnosed before the beginning of registration.

The limitations of the DCO index underline the need for better approaches to assess incompleteness of cancer registration. A variety of alternative proposals to assess incompleteness of cancer registration has been made, a full discussion of which would be beyond the scope of this paper.

A commonly used index is the mortality/incidence ratio (Coleman and Démaret, 1988; Jensen *et al.*, 1991; Parkin *et al.*, 1992). Obviously, this index shares the major limitation of the DCO index, the dependency on the case fatality rate and on the inaccuracy of death certificates.

The limitations of the DCO index outlined in the present paper equally apply to the DCO index (proportion of cases first notified by death certificates), which has been proposed as an alternative measure of incompleteness. The relation of the DCN index and incompleteness depends on case fatality rates and misclassification rates of cancer deaths among patients for whom no primary notification by sources other than the death certificate is made. For these patients, misclassification rates of cancer deaths will typically be somewhat lower than for DCO cases though, since misdiagnoses can be corrected among those patients for whom a follow-back notification by other sources is obtained.

The conceptually simplest and most definitive method to check completeness of cancer registration is to identify a series of cases from an independent source, e.g. lists of probands in clinical trials or other research studies, and to assess how many have been registered (Goldberg *et al.*, 1980; Nwene and Smith, 1982; Mattson *et al.*, 1985; Hunt and Coleman, 1987; Villard-Mackintosh *et al.*, 1988; Swerdlow *et al.*, 1993; Schouten *et al.*, 1993). Such independent lists are often unavailable, however. Therefore, this approach can rarely be employed for routine comparisons of completeness of registration between registries.

Recently, capture–recapture methods have attracted much interest as yet another approach to estimate completeness of disease monitoring (Wittes *et al.*, 1974; Robles *et al.*, 1988; Hilsenbeck *et al.*, 1992; McCarty *et al.*, 1993; Brenner, 1995; Brenner *et al.*, 1995). The principle is to estimate the number of missed cases by the degree of overlap between multiple incomplete sources of case ascertainment. Although it is still too early for definitive judgement, this methodology appears to be promising and should be further developed.

**Acknowledgements**

The author gratefully acknowledges helpful comments from Dr Tim E Aldrich and Dr Leo Schouten on an earlier draft of this manuscript.

**References**

BARCLAY THC AND PHILLIPS AJ. (1962). The accuracy of cancer diagnosis on death certificates. *Cancer*, **15**, 5–9.

BENN RT, LECK I AND NWENE UP. (1982). Estimation of completeness of cancer registration. *Int. J. Epidemiol.* **11**, 362–367.

BRENNER H. (1995). Use and limitations of the capture–recapture method in disease monitoring with two dependent sources. *Epidemiology*, **6**, 42–48.

BRENNER H, STEGMAIER C AND ZIEGLER H. (1995). Estimating completeness of cancer registration in Saarland/Germany with capture-recapture methods. *Eur. J. Cancer* (in press).

COLEMAN MP AND DÉMARET E. (1988). Cancer registration in the European Community. *Int. J. Cancer* **42**, 339–345.

DE FAIRE U, FRIBERG L, LORICH U AND LUNDMAN T. (1976). A validation of cause-of-death certification in 1156 deaths. *Acta Med. Scand.*, **200**, 223–228.

DEVESA SS, POLLACK ES AND YOUNG JL. (1984). Assessing the validity of observed cancer incidence trends. *Am. J. Epidemiol.*, **119**, 274–291.

EDERER F, AXTELL LM AND CUTLER SJ. (1961). The relative survival rate: a statistical methodology. *Natl Cancer Inst. Monogr.*, **6**, 101–121.

FREEDMAN LS. (1978). Variations in the level of reporting by hospitals to a regional cancer registry. *Br. J. Cancer*, **37**, 861–865.

GOLDBERG J, GELFAND HM AND LEVY PS. (1980). Registry evaluation methods: a review and a case study. *Epidemiol Rev.*, **2**, 210–220.

HILSENBECK SG, KURUCZ C AND DUNCAN RC. (1992). Estimation of completeness and adjustment of age-specific and age-standardized incidence rates. *Biometrics*, **48**, 1249–1262.

HUNT K AND COLEMAN MP. (1987). The completeness of cancer registration in follow-up studies – a cautionary note. *Br. J. Cancer*, **56**, 357–359.

JENSEN OM, PARKIN DM, MACLENNAN R, MUIR CS AND SKEET RG. (eds.) (1991). *Cancer Registration: Principles and methods.*, IARC Scientific Publications No. 95. IARC: Lyon.

LOGAN WPD. (1978). Cancer survival statistics international data. *Wld Hlth Stat. Q.*, **31**, 62–73.

McCARTY DJ, TULL ES, MOY CS, KWOH CK AND LAPORTE RE. (1993). Ascertainment corrected rates: applications of capture–recapture methods. *Int. J. Epidemiol.*, **22**, 559–565.

MATTSON B, RUTQVIST LE AND WALLGREN A. (1985). Under-notification of diagnosed cancer cases to the Stockholm cancer registry. *Int. J. Epidemiol*, **14**, 64–69.

NWENE U AND SMITH A. (1982). Assessing completeness of cancer registration in the north-western region of England by a method of independent comparison. *Br. J. Cancer*, **46**, 635–639.

PARKIN DM, MUIR CS, WHELAN SL, GAO Y-T, FERLAY J AND POWELL J. (1992). *Cancer Incidence in Five Continents*, Vol. VI, IARC Scientific Publications No. 120. IARC: Lyon.

PERCY C, STANEK E AND GLOECKLER L. (1981). Accuracy of cancer death certificates and its effect on cancer mortality statistics. *Am. J. Public Health*, **71**, 242–250.

ROBLES SC, MARRETT LD, CLARKE EA AND RISCH A. (1988). An application of capture–recapture methods to the estimates of completeness of cancer registration. *J. Clin. Epidemiol.*, **41**, 495–501.

SCHOUTEN LJ, HÖPPENER P, VAN DEN BRANDT PA, KNOTTNERUS JA AND JAGER JJ. (1993). Completeness of cancer registration in Limburg, the Netherlands. *Int. J. Epidemiol*, **22**, 369–376.

SHANMUGARATNAM K. (1989). Availability and completeness of cancer registration worldwide. *Rec. Res. Cancer Res.*, **114**, 28–33.

SWERDLOW AJ, DOUGLAS AJ, VAUGHAN HUDSON G AND VAUGHAN HUDSON B. (1993). Completeness of cancer registration in England and Wales: an assessment based on 2,145 patients with Hodgkin's disease independently registered by the British National Lymphoma Investigation. *Br. J. Cancer*, **67**, 326–329.

VILLARD-MACKINTOSH L, COLEMAN MP AND VESSEY MP. (1988). The completeness of cancer registration in England: an assessment from the Oxford–FPA contraceptive study. *Br. J. Cancer*, **58**, 507–511.

WITTES JT, COLTON T AND SIDEL VW. (1974). Capture–recapture methods for assessing the completeness of case ascertainment when using multiple information sources. *J. Chron. Dis.*, **27**, 25–36.