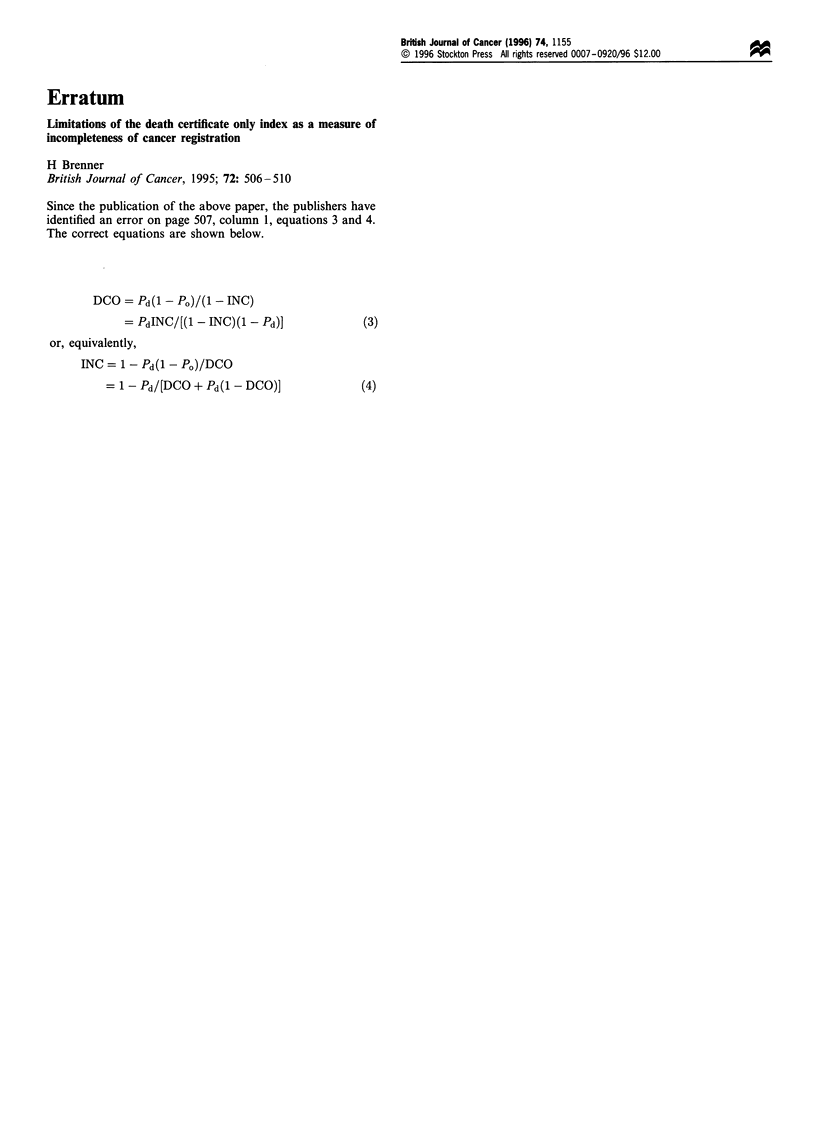# Limitations of the death certificate only index as a measure of incompleteness of cancer registration

**Published:** 1996-10

**Authors:** 


British Journal of Cancer (1996) 74, 1155
© 1996 Stockton Press All rights reserved 0007-0920/96 $12.00

### Erratum

**Limitations of the death certificate only index as a measure of incompleteness of cancer registration**

H Brenner
*British Journal of Cancer*, 1995; **72:** 506-510

Since the publication of the above paper, the publishers have identified an error on page 507, column 1, equations 3 and 4. The correct equations are shown below.

$$\begin{aligned} \mathrm{DCO} &= P_d(1 - P_o)/(1 - \mathrm{INC}) \\ &= P_d\mathrm{INC}/[(1 - \mathrm{INC})(1 - P_d)] \end{aligned} \quad (3)$$

or, equivalently,

$$\begin{aligned} \mathrm{INC} &= 1 - P_d(1 - P_o)/\mathrm{DCO} \\ &= 1 - P_d/[\mathrm{DCO} + P_d(1 - \mathrm{DCO})] \end{aligned} \quad (4)$$